# Functional classification of CATH superfamilies: a domain-based approach for protein function annotation

**DOI:** 10.1093/bioinformatics/btv398

**Published:** 2015-07-02

**Authors:** Sayoni Das, David Lee, Ian Sillitoe, Natalie L. Dawson, Jonathan G. Lees, Christine A. Orengo

**Affiliations:** Institute of Structural and Molecular Biology, UCL, Darwin Building, Gower Street, WC1E 6BT, UK

## Abstract

**Motivation:** Computational approaches that can predict protein functions are essential to bridge the widening function annotation gap especially since <1.0% of all proteins in UniProtKB have been experimentally characterized. We present a domain-based method for protein function classification and prediction of functional sites that exploits functional sub-classification of CATH superfamilies. The superfamilies are sub-classified into functional families (FunFams) using a hierarchical clustering algorithm supervised by a new classification method, FunFHMMer.

**Results:** FunFHMMer generates more functionally coherent groupings of protein sequences than other domain-based protein classifications. This has been validated using known functional information. The conserved positions predicted by the FunFams are also found to be enriched in known functional residues. Moreover, the functional annotations provided by the FunFams are found to be more precise than other domain-based resources. FunFHMMer currently identifies 110 439 FunFams in 2735 superfamilies which can be used to functionally annotate > 16 million domain sequences.

**Availability and implementation:** All FunFam annotation data are made available through the CATH webpages (http://www.cathdb.info). The FunFHMMer webserver (http://www.cathdb.info/search/by_funfhmmer) allows users to submit query sequences for assignment to a CATH FunFam.

**Contact:** sayoni.das.12@ucl.ac.uk

**Supplementary information:** Supplementary data are available at *Bioinformatics* online.

## 1 Introduction

The rapid increase in sequencing and structural genomics initiatives have provided us with a wealth of data to unravel the complex sequence, structure and function relationships in proteins. However, < 1.0% of this data are functionally annotated from experimental studies (UniProtKB, January 2015).

The most commonly used methods for protein function annotation often exploit a sequence or structure homology search of a query protein against a protein database, although more sophisticated approaches which also exploit heterogeneous data (e.g. gene expression and protein–protein interaction data, etc.) and combine this diverse information using machine-learning approaches have also been developed. [For a review of current approaches, see Radivojac *et* *al*. (2013)]. It has been observed that proteins sharing high sequence or structural similarity can often evolve different (sub-)functions (Bashton and Chothia, 2007; Glasner *et al.*, 2006; Hannenhalli and Russell, 2000). These functionally diverse relatives perform distinct functions even though they may share a general functional feature, e.g. for enzymes this may be a common step along their reaction pathways. Such groups of homologous proteins sharing the same function will be referred to as functional families hereafter.

Classification of protein space into functional families is useful for annotating uncharacterized sequences by inheriting annotations from characterized sequences in the family the query sequence matches best. The identification of functional families and characterization of their functional sites is also important for understanding how function is modulated during evolution by sequence and structural changes in diverse homologous groups (Hannenhalli and Russell, 2000).

Protein-based family resources like PANTHER (Mi *et* *al.*, 2013), TIGRFAMs (Haft *et* *al.*, 2013), HAMAP (Lima *et* *al.*, 2009) and specialized databases like Structure-Function Linkage Database (SFLD) (Akiva *et* *al.*, 2014), TEED (Widmann *et* *al.*, 2010) provide manually curated functional grouping of protein sequences which are limited by low sequence coverage. Using more automated approaches, ProtoNet (Rappoport *et* *al.*, 2014) provides an automatic classification of similar proteins which are further sub-classified into clusters using an information-theoretic protocol based on available annotations. Another automated protein family resource, PhyloFacts (Krishnamurthy *et* *al.*, 2006) provides a collection of protein families which have been further classified into subfamilies by the SCI-PHY algorithm which uses Bayesian and information-theoretic measures to construct a hierarchical phylogenetic tree and determine an optimal cut of the tree into families (Sjolander, 1998).

When global protein homology searches fail, i.e. an uncharacterized protein cannot be assigned to any characterized whole protein families, function can perhaps be better understood by analysing its domain components and finding functionally characterized homologs for each domain. Exploiting this approach, a ‘domain grammar’, (Dessailly *et* *al.*, 2009) which exploits information from a protein domain resource, can be used to describe protein function.

There are many protein domain resources which provide classifications of protein domains based on either sequence (e.g. Pfam) or structure [e.g. CATH (Sillitoe *et* *al.*, 2015) and SCOP (Murzin *et* *al.*, 1995)]. Pfam (Sonnhammer *et* *al.*, 1997) is the most comprehensive and widely used database of protein domain families. The Pfam version 27.0 release comprises 14 831 manually curated Pfam-A families which provides ∼80% coverage of the UniProtKB sequence space (Finn *et* *al.*, 2014). The Funshift (Abhiman and Sonnhammer, 2005) database classifies Pfam version 12.0 families into subfamilies using the SCI-PHY algorithm and provides analysis of function shifts of subfamilies within a Pfam family (Brown *et al.*, 2007; Sjolander, 1998).

The protein structure classification databases CATH (Sillitoe *et* *al.*, 2015) and SCOP (Murzin *et* *al.*, 1995) extracts structural information from the Protein Data Bank (PDB) and classifies protein domains into evolutionary-related superfamilies based on their evolutionary origin, exploiting structural data to bring together very distant evolutionary-related domains in a superfamily. Additionally, Gene3D (Lees *et* *al.*, 2014) and SUPERFAMILY (de Lima Morais *et* *al.*, 2011) provide broader sequence coverage for each superfamily in CATH and SCOP, respectively.

SCOP sub-classifies its superfamilies into manually curated families. However, these have been found to more closely resemble taxonomic groups rather than functional groups (Pethica *et* *al.*, 2012). A few protein resources like InterPro (Mitchell *et* *al.*, 2015) and the Conserved Domain Database (CDD) (Marchler-Bauer *et* *al.*, 2015) also combine multiple protein domain family databases like Pfam, SMART, TIGRFAM among others, providing higher sequence coverage compared with individual resources. Thus, it can be clearly seen that although, there are several well-established resources for classifying homologous sequences into protein or domain families, most have not traditionally sub-classified relatives according to functional similarity using automated approaches, because of the complex nature of this task.

Sequences in the CATH-Gene3D resource have been classified into functional families or FunFams by cutting a hierarchical tree of sequence relatives produced by a clustering algorithm, GeMMA (Lee *et* *al.*, 2010) at a generic threshold. This was later improved by the Domain Family Exploration (DFX) algorithm which used function annotation data from the Gene Ontology (GO) (Ashburner *et* *al.*, 2000) to sub-classify the superfamilies into FunFams (Rentzsch and Orengo, 2013). However, due to the paucity of the GO terms and annotation biases existing in the GO (Schnoes *et* *al.*, 2013), new approaches for functionally classifying CATH superfamilies have been explored which exploit sequence patterns, and are unaffected by the limitations of GO.

Here, we present a new and much more accurate method for functional classification of CATH superfamilies into FunFams that can be used for protein function annotation and prediction of functionally important residues. The new protocol, FunFHMMer, determines an optimal cut of a hierarchical clustering tree of sequence relatives within a given superfamily by calculating a novel functional coherence index based on conserved positions and specificity-determining positions (SDPs) in sequence alignments. Prediction of SDPs has been used in the past to generate functional groups for a number of selected protein superfamilies (Costa *et* *al.*, 2013; Mazin *et al.*, 2010; Rausell *et* *al.*, 2010; Reva *et* *al.*, 2007). However, none of these approaches has been used for large-scale sub-classification of all known protein domain superfamilies. Moreover, these methods also require an accurate multiple-sequence alignment of all sequences as a starting point which can lead to erroneous sub-classification of very large and diverse superfamilies.

The functional purity of the classification has been validated by known functional information. For example, the predicted conserved sites of the FunFams are found to be enriched in experimentally characterized functional residues and the functional annotations provided by FunFams are found to be more precise compared with those generated by other domain-based resources. Furthermore, the FunFHMMer prediction protocol is able to provide functional annotations for nearly >16 million domain sequences in UniProtKB and Ensembl.

## 2 Methods

### 2.1 Superfamily sequence clustering

For each CATH-Gene3D superfamily, the sequences are first pre-clustered at 90% sequence identity into S90 clusters using CD-HIT (Fu *et* *al.*, 2012). All sequences are associated with GO annotations from UniProt-GOA (dated May 2013). Any S90 cluster which lacks at least one sequence with high-quality GO annotations is removed, as each family must contain at least one relative with reliable function annotations for annotation transfer to uncharacterized sequences. High-quality GO annotations were considered to be those which were Inferred from Electronic Annotations (IEAs) in Swiss-Prot made by EC2GO or Swiss-Prot Keyword2GO mapping methods as well as annotations experimentally inferred or curated (non-IEA) in UniProtKB (Rentzsch and Orengo, 2013; Škunca *et al.*, 2012). Fragments (< 80% of the average sequence length) are then removed from the remaining clusters. These clusters form the starting clusters for the profile-based agglomerative clustering algorithm, GeMMA (Lee *et* *al.*, 2010). GeMMA exploits COMPASS (Sadreyev and Grishin, 2003) to compare the profiles derived from the multiple sequence alignments (MSAs) of clusters present at each stage of the clustering. At each iteration, the cluster profiles matching above a threshold are merged and profiles are generated for the new clusters. These iterations continue giving a hierarchical clustering tree built from the leaf nodes to the root, till a single cluster remains.

### 2.2 FunFHMMer algorithm

**Fig. 1.**
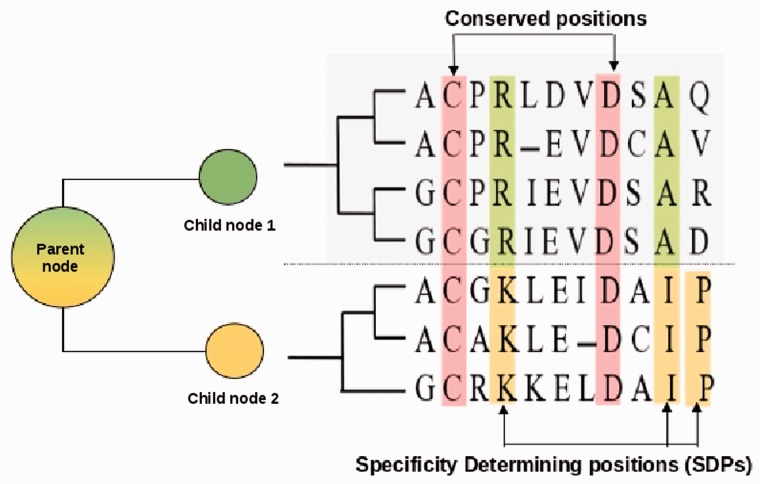
Use of SDPs by FunFHMMer to infer functional coherence of cluster alignments. The coloured circles represent the node sequence clusters and each colour denotes a unique function. The schematic representation of the parent node MSA and the child nodes MSA is shown along with the phylogenetic tree. Child nodes are separated by a dashed line. Conserved positions in the MSA are shown in red and the SDPs are shown in green or yellow for different child nodes

The FunFHMMer algorithm is an automated classification protocol that determines the optimal cut of this ‘bottom-up’ hierarchical clustering tree (Supplementary Fig. S1) to identify FunFams in protein domain superfamilies. It identifies highly conserved positions and SDPs in cluster alignments (Fig. 1) and calculates a novel Functional Coherence index (*FC*) for each parent node in the tree. This value is to determine whether the child nodes should be merged.

The conserved positions in all sequences in an alignment are generally important for the stability, folding or function of the protein domain. In contrast, SDPs or positions conserved within groups of sequences which share a specific (sub-)function, are usually involved in functional divergence (Abhiman and Sonnhammer, 2005; Rausell *et* *al.*, 2010).

#### 2.2.1 Functional coherence index (FC)

The analysis of functional coherence of a parent node takes into account the following parameters:

##### 1. Information content of MSAs

An MSA which comprises evolutionary distant relatives is considered to be highly informative. The diversity of residue conservation of positions in informative MSAs not only helps to prevent bias but also provides more discriminating conservation scores (Bartlett *et* *al.*, 2002).

FunFHMMer calculates Diversity of Position Scores (DOPS) for MSAs using Scorecons (Valdar, 2002). DOPS is 0 if all positions in an alignment have the same conservation score and 100 when no two positions have the same conservation score. For our analysis, we have considered any alignment with a DOPS > 70, as sufficiently diverse (Dessailly *et* *al.*, 2013). For less diverse alignments, any MSA analysis will have a higher probability of predicting false positives (false SDPs, in this case) as a result of less discriminatory conservation scores. To account for this, a DOPS factor $\left(D_{f}\right)$ is used, where $D_{f}=1$, if both groups have DOPS > 70 and $D_{f}=0$, if either sub-group have DOPS < 70.

##### 2. Proportion of predicted SDPs in an MSA

FunFHMMer uses GroupSim (Capra and Singh, 2008) to predict SDPs in MSAs of parent nodes in the clustering tree. GroupSim takes an MSA containing pre-defined groups (clusters associated with two child nodes in the tree) as input and calculates a prediction score (*G_s_*) for each column in the alignment. *G_s_* ranges from 0 to 1 where higher scores indicate a higher probability for a column in an alignment to be an SDP.

In order to discriminate easily between conserved positions and SDPs using *G_s_*, a threshold was identified that distinguishes between these positions using a benchmark dataset generated by Chakraborty and Chakrabarti (2015) (Supplementary Section S2). Henceforth, we defined all positions with $\left(G_{s}\leq 0.3\right)$ as conserved positions and those with 0.7 < $G_{s}\;\leq \;1$ as SDPs in our subsequent analysis of parent nodes.

For each parent node being analysed by FunFHMMer, its child nodes form the predefined groups for GroupSim. The number of SDPs $\left(N_{\text{sdp}}\right)$ and the number of conserved positions $\left(N_{c}\right)$ are calculated from the Groupsim prediction scores for the parent node MSA. Whether two child nodes are merged depends on the ratio of SDPs to conserved positions in the parent node MSA (*R*_sdp_). However, optimization trials showed that this ratio $\left(R_{\text{sdp}}\right)$ needed to be adjusted if one or both the child nodes had a low DOPS score. The *R*_sdp_ ratio for a parent node is therefore calculated as:

(1) $$R_{\text{sdp}}=\;D_{f}\left(\frac{N_{\text{sdp}}}{N_{c}+N_{\text{sdp}}}-0.2\right)+(1-D_{f})\left(\frac{N_{\text{sdp}}}{N_{c}}-1\right)$$

where *D_f_* is the DOPS factor of the MSA, *N_sdp_* is the number of SDPs, *N_c_* is the number of conserved positions in the MSA. For more details on how Equation (1) was derived, see Supplementary Section S3.

##### 3. Gaps in an MSA

A large number of gaps in a parent node alignment would indicate that the group alignments are of different lengths. The coherence index uses a gap factor *f*_gap_ which is dependant on the number of non-gapped $\left(N_{\text{nongap}}\right)$ and gapped positions $\left(N_{\text{gap}}\right)$ in the alignment. Such that, $f_{\text{gap}}=$ 0, if $N_{\text{nongap}}\;>\;N_{\text{gap}}$ and $f_{\text{gap}}=$ 1, if $N_{\text{nongap}}\;\leq \;N_{\text{gap}}$ in MSA.

Bringing together all the above parameters, the Functional Coherence index (*FC*) is calculated using the empirical formula described later (Equation 2), where a coherence index of 1 indicates functional coherence of the parent node and 0 indicates that functionally diverse child nodes have been merged to form the parent node.

(2) $$FC=\;\{\begin{array}{cc} 1 & \text{if}\;R_{\text{sdp}}.f_{\text{gap}}\leq 0\\ 0 & \;\text{if}\;R_{\text{sdp}}.f_{\text{gap}}>0 \end{array}$$

where, *R*_sdp_ is the SDP ratio (Equation 1) and *f*_gap_ is the gap factor.

The functional coherence index is used to ensure that only functionally related clusters are merged. The resulting clusters of the tree form the functional families (FunFams) for a protein domain superfamily. The workflow for the FunFHMMer algorithm is shown in Supplementary Figure S4. Once the functional families have been identified sequence patterns (hidden Markov models, HMMs) are derived for each and used to identify further relatives in UniProtKB.

### 2.3 FunFam model generation and mapping of family relatives

For each FunFam in a superfamily, an alignment is generated using MAFFT (Katoh *et* *al.*, 2002) and a profile HMM is built using HMMER3 (Eddy, 2010). A model-specific inclusion threshold score is then determined for each family model by choosing the lowest HMM bit score obtained by scanning all the sequences from which a model was built, against the model itself. After this, all sequences from Gene3D not clustered into an S90 cluster at the start of clustering, are scanned against the family models and a sequence is accepted as a new member of a FunFam if it exceeds the inclusion threshold score of the respective family model.

### 2.4 Identification of functionally important residues in FunFams

Ideally, FunFams are groups of protein domains with a high probability of sharing the same function(s) and therefore the functionally important residues (e.g. catalytic residues, ligand-binding residues) in a family are also expected to be highly conserved. For FunFams with sufficient information content in their MSA, residue conservation scores are calculated for each position in the alignment using Scorecons (Valdar, 2002). Scorecons scores range from 0 to 1 and residues having scores  ≥ 0.7 are considered to be highly conserved (Dessailly *et* *al.*, 2013).

Overlaps between conserved positions in FunFams and known catalytic residues taken from the Catalytic Site Atlas (CSA) (Porter *et* *al.*, 2004) were evaluated using enrichment tests adapted from Dessailly *et* *al.* (2013). For each FunFam, enrichment values were calculated as the difference between the proportion of conserved residues that are catalytic and the proportion of all residues that are catalytic. The enrichment values were averaged for each superfamily and an unpaired, one-sided Wilcoxon rank sum test (Kruskal, 1957) was run on the averaged values using the wilcox.test function in R (Team, 2014). This test assessed a *P*-value for the null hypothesis that the proportion of conserved catalytic residues is the same as the proportion of unconserved functional residues i.e. the median enrichment value is zero.

### 2.5 Functional annotation of uncharacterized sequences

**Fig. 2.**
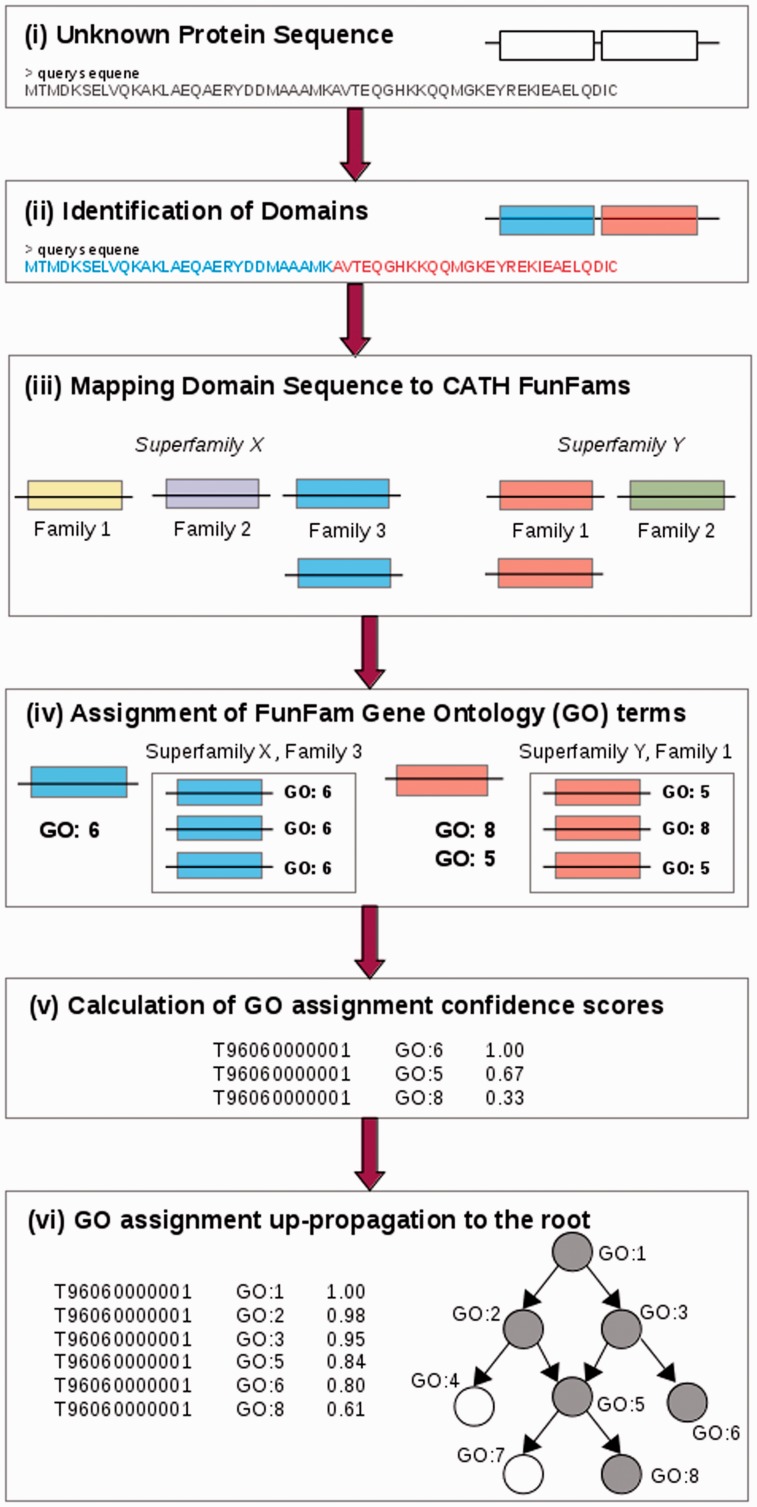
Function prediction using CATH FunFams. Workflow for making function predictions using CATH Functional Families

Uncharacterized protein sequences are scanned against a library of HMMs of CATH superfamilies and domain regions assigned to superfamilies using DomainFinder3 (Yeats *et* *al.*, 2010) [Fig. 2(ii)]. The domain sequences are then scanned against the CATH FunFam models for the given superfamily using HMMER3 (Eddy, 2010) and mapped to their most likely FunFam i.e. the model matched with the highest HMM score, [Fig. 2(iii)] if it achieves the inclusion threshold score of the respective family model.

The GO term annotations of that FunFam are then transferred to the query sequence in a probabilistic manner which is calculated as the annotation frequency of a particular GO term among the seed sequences of the FunFam [Fig. 2(iii)–(iv)]. The GO term confidence scores are subsequently propagated up the GO hierarchy (Fig. 2(vi)]. Finally, the non-redundant set of constituent domain GO term assignments for each domain region in the protein sequence, each GO term retaining its highest confidence score, together make up the function predictions for the whole-protein.

### 2.6 Assessment of functional purity of FunFams

To assess whether sub-classifying the domain data in CATH-Gene3D into FunFams by FunFHMMer improved the functional purity of the FunFams and the ability to use them to transfer functional annotation, we performed the following tests: (i) assessment of the quality of functional classification using known functional information (EC numbers) and (ii) analysis of the predictive power of CATH-Gene3D FunFams using a UniProtKB/Swiss-Prot rollback assessment. In these, we compared FunFHMMer against our previous functional classification method, DFX and other domain-family classifications i.e. Pfam, SUPERFAMILY and CDD. The domain families and superfamilies in these resources have not been explicitly classified according to function and therefore, the only purpose of including them in the assessment was to determine whether there was any benefit in function annotation transfer from sub-classification of the CATH-Gene3D resource into FunFams.

#### 2.6.1 Quality of functional classification based on EC numbers

This test (referred to as the EC assessment hereafter) was used to analyse the performance of protein classifications in distinguishing between sequence relatives having different EC numbers as consistency of EC numbers across a group of sequence relatives is clearly indicative of functional purity. The FunFams generated by FunFHMMer for both CATH superfamilies and Pfam-A families were assessed as were DFX FunFams, CATH superfamilies, Pfam-A families, superfamilies in SUPERFAMILY and families in SUPERFAMILY. Although CATH, Pfam and SUPERFAMILY are not publicized as functional classifications, these resources are frequently used for functional annotation of query sequences.

The EC annotations of all FunFam sequences in CATH were extracted from UniProtKB (dated February 2013) but we only considered those which had a four-digit EC number associated with the whole protein. These sequences were mapped to the different protein classifications used in the assessment and the number of different unique EC numbers per family or superfamily was analysed.

#### 2.6.2 UniProtKB/Swiss-Prot rollback assessment

A CAFA-style assessment was generated by rolling back the UniProtKB/Swiss-Prot database dated November 2013 to May 2013 (6 months before). The assessment comprised well-annotated sequences which did not have any reported GO terms (having GO evidence codes: EXP, IDA, IMP, IGI, IEP, TAS or IC) in the Molecular Function Ontology (MFO) in the May 28, 2013 version of UniProtKB/Swiss-Prot, but had MFO annotations associated with them in the November 28, 2013 version. This resulted in a dataset of 1945 proteins. The distribution of leaf term (MFO) annotations of the assessment proteins is shown in Supplementary Figure S5. Sequence MD5 (a 32 character hexadecimal number) of the query sequences were used to map sequences between databases (Smith *et* *al.*, 2005). Sequences were scanned against FunFams generated by DFX and FunFHMMer to predict functions for this dataset using the protocols described earlier and high-quality MFO annotations (Section 2.1 for details) available up to May 2013. The functional annotations assigned by FunFams generated by FunFHMMer were compared with the annotations provided by Pfam (version 27.0) and CDD (version 3.10) family matches (Supplementary Section S5.2). Pfam and CDD were chosen for the assessment as Pfam is the most comprehensive manually curated domain-based resource which is widely used by biologists for functional annotation and CDD is a widely used comprehensive protein resource that integrates multiple curated protein and protein domain family databases such as Pfam, SMART, COGs (Cluster of Orthologous Groups), TIGRFAMs, NCBI Protein Clusters and NCBI Curated Domains. Each classification protocol was evaluated only on the subset of the assessment dataset for which it predicted at least one GO annotation.

The performance of the functions predicted by different classification methods was measured using Precision-Recall (PR) graphs by comparing the maximum F-measure $\left(F_{\text{max}}\right)$ (harmonic mean between precision and recall) values in the same manner as used by Radivojac *et* *al.* (2013) in the CAFA (Critical Assessment of protein Function Annotation) function prediction assessment (Supplementary Section S5.3).

### 2.7 Generation of CATH superfamily networks

CATH superfamily networks are constructed in which FunFams are represented by nodes and the edge distances correspond to the sequence similarity between the FunFams, assessed using Profile Comparer (PRC) (Madera, 2008) which compares the sequence profiles derived from the multiple alignments of the families. The networks are visualized in the prefuse force-directed layout with edges weighted by the PRC score using the Data-Driven Documents (D3) JavaScript library (Bostock *et* *al.*, 2011).

## 3 Experiments and results

### 3.1 Generation of CATH FunFams

FunFHMMer was used to generate a new set of FunFams for CATH v4.0 (Sillitoe *et* *al.*, 2015). A total of 110 439 FunFams were generated by FunFHMMer for 2735 CATH superfamilies. By scanning UniProtKB sequences against CATH-Gene3D and FunFam HMMs (see Section 2.3 in Methods), > 16 million sequences can be mapped to the FunFams and annotated with functional information. All FunFam data are made available through the CATH webpages which provides a listing of FunFams within each superfamily. For each FunFam, visualization of the MSA is provided, which is also available for download.

FunFHMMer was also used to generate FunFams for 14 831 Pfam-A families giving 172 211 Pfam-A FunFams. In the text below, Pfam families that have not been sub-classified using FunFHMMer are referred to as Pfam (native) families. FunFams were also generated for the CATH superfamiles using the DFX algorithm which resulted in 26 760 DFX FunFams.

### 3.2 Functionally important residues are highly conserved in FunFams

The conserved residues in FunFam alignments (Section 2.4) were found to be significantly enriched in known catalytic residues, i.e. FunFams have a greater proportion of conserved catalytic residues in comparison to unconserved catalytic residues (*P*-value < 3.64E-51). Moreover, the FunFams were also found to have a larger proportion of conserved catalytic residues compared with our previous classification (Supplementary Section S6). The highly conserved residues in the FunFams identified by FunFHMMer are highlighted on a representative 3D-structure for the FunFam and can be viewed on the CATH webpages.

### 3.3 Assessment of functional purity of FunFams

#### 3.3.1 Quality of functional classification using EC numbers

The EC assessment dataset (Section 2.6.1) in CATH, consisting of 670 128 sequences, mapped to 1664 CATH superfamilies, 33 668 CATH FunFams generated by FunFHMMer, 9215 CATH FunFams generated by DFX, 4856 Pfam (native) families, 24 789 Pfam FunFams generated by FunFHMMer, 1187 superfamilies in SUPERFAMILY and 2509 families in SUPERFAMILY.

**Fig. 3.**
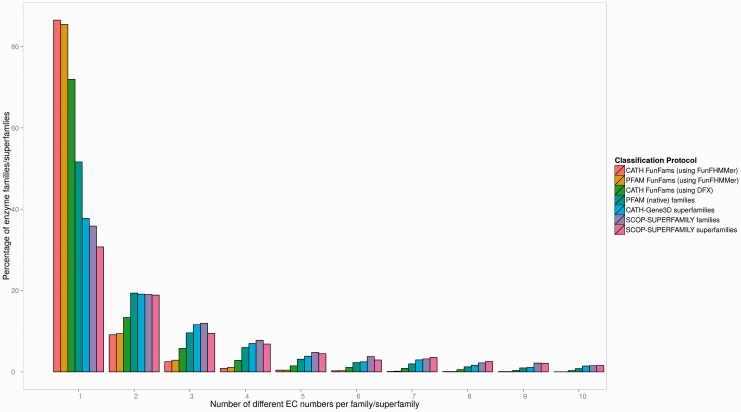
EC number variation across protein classifications. Percentage of families or superfamilies having a certain number of EC terms for each of the domain-based protein classifications

Figure 3 shows the proportions of different sequence groupings (families or superfamilies) generated by the above-mentioned protein classifications having relatives with one or many different EC numbers. The figure has been truncated to show the proportion of families or superfamilies, up to a maximum of 10 different ECs per sequence grouping by a classification protocol. The highest proportion of families found to have only one EC number associated with them were CATH FunFams (86.5%) and the Pfam FunFams (85.5%) generated by FunFHMMer, followed by CATH FunFams (71.9%) generated by DFX, Pfam (native) families (51.6%), CATH superfamilies (37.7%), families in SUPERFAMILY (35.8%) and superfamilies in SUPERFAMILY (30.7%). This illustrates that the FunFams generated by FunFHMMer provide a more functionally coherent grouping of protein sequences than the other domain classifications. Moreover, it also shows that the FunFHMMer classification protocol is not limited in its use to CATH but can also be used to sub-classify other widely used domain-based classification resources such as Pfam.

Furthermore, we also assessed the quality of our functional sub-classification by comparing functional assignments against the renowned SFLD which has been used in benchmarking other functional classifications (Brown *et* *al.*, 2007 and Lee *et* *al.*, 2010). Results are given in the Supplementary Section S7 which showed that FunFHMMer provides functional families that correspond well with the manually curated SFLD families.

#### 3.3.2 UniProtKB/Swiss-Prot rollback assessment

**Fig. 4.**
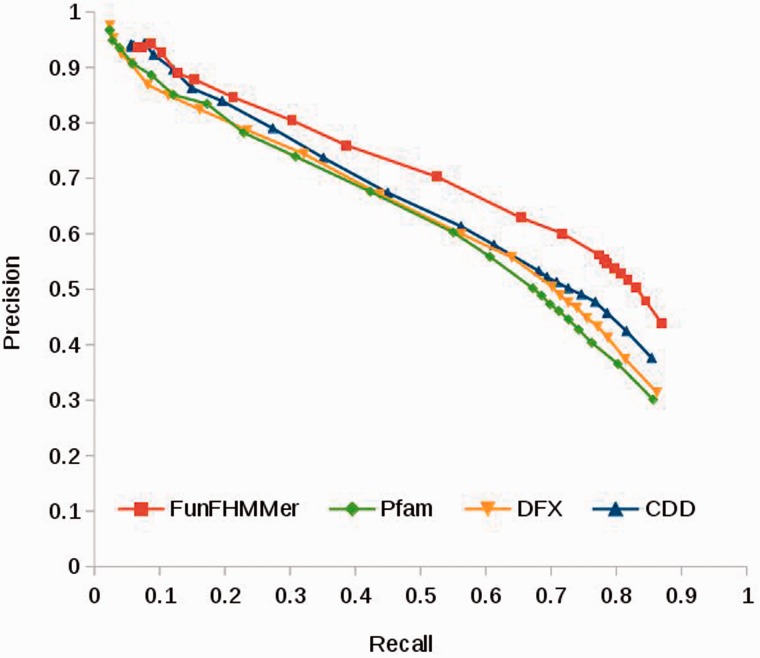
UniProt rollback assessment. Performance of FunFHMMer protocol on the UniProtKB/Swiss-Prot rollback assessment dataset compared with functional annotations predicted by DFX protocol, Pfam (native) family and CDD family assignments

The PR graph in Figure 4 shows the performance of FunFams generated by FunFHMMer in predicting functions for the rollback assessment compared with functions predicted by Pfam (native) families, CDD families and DFX FunFams at different confidence score thresholds ranging from 0 to 1. Pfam provides predictions for the highest number of sequences (Coverage (C) = 86.5%) in the dataset followed by DFX (C = 75.8%), CDD (C = 74.7%) and FunFHMMer (C = 74%).

From Figure 4, we observe that all the methods perform competitively. For predictions with high confidence scores (thresholds > 0.95), Pfam (native) and DFX families i.e. broader groupings of protein sequences can predict functions with higher precision than CDD and FunFHMMer. However, for all other predictions with lower confidence scores (thresholds < 0.95), CDD and FunFHMMer perform better with respect to both precision and recall. For this dataset, FunFHMMer gives the highest maximum F-measure (*F*_max_ = 0.653) than the other family resources (CDD *F*_max_ = 0.598; DFX *F*_max_ = 0.595; Pfam *F*_max_ = 0.581). The relative performance of the methods was the same for hard targets of the assessment i.e. those proteins which do not have any functionally annotated relatives with sequence identity > 50% (Supplementary Section S5.4). FunFHMMer also shows better performance (higher *F*_max_ value) in predicting protein functions compared with DFX which confirms that, as expected, improved functional sub-classification of CATH superfamilies also improves protein function prediction and that the purity of the FunFams can have a significant impact on their performance in functional annotation of uncharacterized sequences.

### 3.4 Visualization of FunFam relationships within superfamilies

**Fig. 5.**
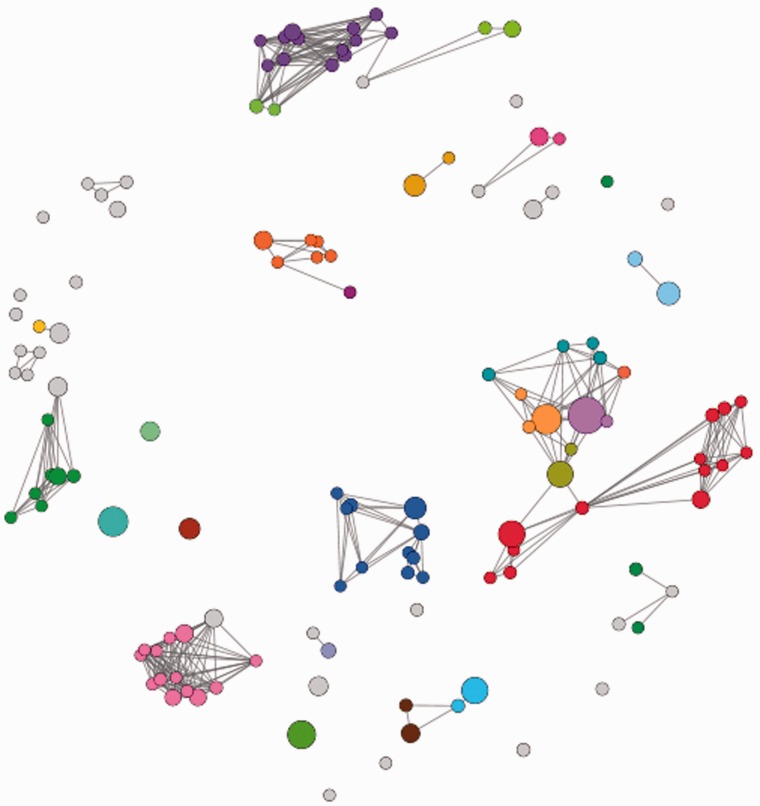
Network representation of the HUP Superfamily (CATH 3.40.50.620) showing available functional annotations in FunFams. The coloured nodes indicate FunFams annotated with different EC numbers and the grey nodes indicate FunFams without any Enzyme Commission (EC) annotation which include non-enzymes

The superfamily networks can be very useful in providing a comprehensive summary of the relationships between FunFams in a superfamily. For example, Figure 5 shows the network for the large and diverse HUP superfamily (High-signature proteins, UspA and PP-ATPase, CATH 3.40.50.620) showing only FunFams with high information content. Study of such superfamily networks can provide useful insights into the evolution of functional diversity within the superfamily. All superfamily networks can be viewed on the CATH web pages.

## 4 Conclusion

The CATH-Gene3D resource provides a comprehensive classification of structure and sequence domains into 2735 structure-based superfamilies. We have developed a novel method (FunFHMMer) for functional classification of these superfamilies. The aim of this classification is manifold. For example, to improve our understanding of the sequence and structure mechanisms of functional divergence within a superfamily during evolution and to improve the functional annotation of uncharacterized protein domain sequences assigned to an annotated functional family within the superfamily. To our knowledge no other such comprehensive functional classification of domain sequences, linked to structural data, exists.

Here, we show that our novel functional classification protocol has resulted in FunFams that are significantly more functionally pure than in our previous classification reported in 2011 (DFX, Rentzsch and Orengo, 2013). This was demonstrated using an assessment protocol based on the EC classification and the manually curated SFLD superfamilies. We also demonstrate that this enhanced functional purity translates into better performance in functional prediction for uncharacterized sequences using a UniProtKB/Swiss-Prot rollback assessment.

FunFHMMer clearly generates more functionally pure families than other domain-based resources and outperforms DFX in protein function prediction as assessed by the UniProtKB/Swiss-Prot rollback assessment. Moreover, the performance of FunFHMMer in the preliminary results of CAFA 2 (2013–2014) have been very encouraging. FunFHMMer, which only uses domain information, was featured among the top five among 110 automatic function prediction methods (some of which use information from heterogenous data sources for making predictions) in predicting biological process GO terms and among the top 10 in predicting molecular function terms. CAFA 2 results can be accessed from: https://github.com/idoerg/CAFA2-results. The CAFA assessment protocol provides independent validation that the FunFams in CATH-Gene3D are of reasonable functional purity and valuable for providing functional annotations for novel, uncharacterized sequences.

CATH currently identifies 110 439 FunFams and for the most populated of these (having high information content), accounting for 72% of CATH-Gene3D sequences, residues implicated in functional sites can be predicted. Our assessment of functional purity of the FunFams has shown that our new families can be used to identify a higher proportion of known functional residues associated with a particular functional grouping than our previous method DFX. A web server has been set up to allow users to submit query sequences for assignment to a CATH FunFam, where possible. The server (http://www.cathdb.info/search/by_funfhmmer) takes a protein sequence in the FASTA format as input and scans it against the CATH FunFam HMMs. The FunFam matches for the constituent domains of the query sequence are reported when they achieve the inclusion threshold score of the corresponding FunFam HMM. Information on the highly conserved residues identified for those CATH FunFams having MSAs with sufficient information content, is also available on the CATH-Gene3D website (Lees *et al.*, 2014; Sillitoe *et* *al.*, 2015). These positions are highlighted on a representative 3D-structure for the FunFam, where available.

We also provide network visualizations of the relationships between FunFams within each superfamily. These images provide valuable insights into the functional diversity across a superfamily and reveal where particular functions dominate the superfamily. Therefore, CATH FunFams are useful for analysing the variation in functions across a superfamily and since functional sites can be identified for many FunFams, they allow a structurally informed analysis of the mechanisms of this divergence.

In summary, we have shown that a novel approach to sub-classifying FunFams in CATH-Gene3D, based on the difference in SDPs between functional groups is able to separate FunFams exploiting different conserved residues to perform their functional properties. This approach provides a domain classification that is able to provide accurate functional annotations than broader groupings of relatives.

## Supplementary Material

Supplementary Data
