# Functional classification of CATH superfamilies: a domain-based approach for protein function annotation

**DOI:** 10.1093/bioinformatics/btw473

**Published:** 2016-07-31

**Authors:** Sayoni Das, David Lee, Ian Sillitoe, Natalie L. Dawson, Jonathan G. Lees, Christine A. Orengo

*Bioinformatic*s (2015) **31**(21):3460–3467.

Author, Sayoni Das, would like to report a typographical error in Equation (2) in the above article.

Equation (2), on page 3462, should read as follows:

FC=1,if Rsdp+fgap<00,if Rsdp+fgap⩾0

