# Supplementary material for: Functional classification of CATH superfamilies: a domain-based approach for protein function annotation
Source: Bioinformatics. 2015 Jul 2;31(21):3460–7. doi: 10.1093/bioinformatics/btv398 (PMC4612221; doi:10.1093/bioinformatics/btv398)
Supplement: Supplementary Data [file supp_31_21_3460__index.html]

Functional classification of CATH superfamilies: a domain-based approach for protein function annotation — Functional classification of CATH superfamilies: a domain-based approach for protein function annotation — Supplementary Data 

# Functional classification of CATH superfamilies: a domain-based approach for protein function annotation

## Supplementary Data

files

- Supplementary Data - pdf file
