# Supplementary material for: Functional classification of CATH superfamilies: a domain-based approach for protein function annotation
Source: Bioinformatics. 2015 Jul 2;31(21):3460–7. doi: 10.1093/bioinformatics/btv398 (PMC4612221; doi:10.1093/bioinformatics/btv398)
Supplement: Supplementary Data [file supp_btv398_Supplementary_Data.pdf]

Supplementary Data:
Functional classification of CATH superfamilies: a domain-based approach for protein function annotation

Sayoni Das, David Lee, Ian Sillitoe, Natalie L. Dawson,
Jonathan G. Lees and Christine A. Orengo

Institute of Structural and Molecular Biology, UCL, Gower Street, WC1E 6BT, UK

1 Optimal cut of hierarchical clustering tree

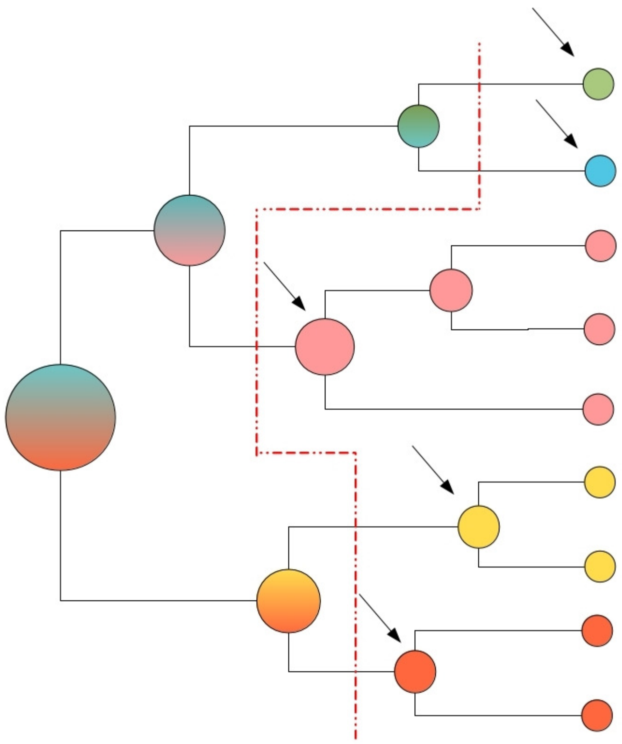

Supplementary Fig. 1: Ideal partitioning of GeMMA tree. The coloured circles represent the sequence clusters and each colour denotes a unique function and the red dashed line denotes the optimal cut of the GeMMA tree. The ideal functional families are indicated by arrows.

2 Differentiating between conserved positions and SDPs in MSAs using GroupSim.

A threshold was identified to distinguish easily between conserved positions and SDPs in MSAs using the GroupSim prediction scores (G_s) using a benchmark dataset generated by Chakraborty and Chakrabarti (2014) (previously used for showing that GroupSim outperforms most other methods for SDP prediction). However, ranked predictions were used in this work and no thresholds were identified.

The benchmark consisted of 20 manually-curated protein family alignments with well identified groups and SDPs (Chakrabarti *et al.*, 2007; Chakraborty and Chakrabarti, 2014). GroupSim was run on each alignment in the benchmark and the range of scores for conserved positions and for SDPs was determined. In Supplementary Figure 1 we see that the majority of conserved positions were found to have ($G_s \leq 0.3$) and the majority of SDPs were found to be in the range $0.7 < G_s \leq 1$. Henceforth, we defined all positions with ($G_s \leq 0.3$) as conserved positions and those with $0.7 < G_s \leq 1$ as SDPs in our subsequent analysis of parent nodes.

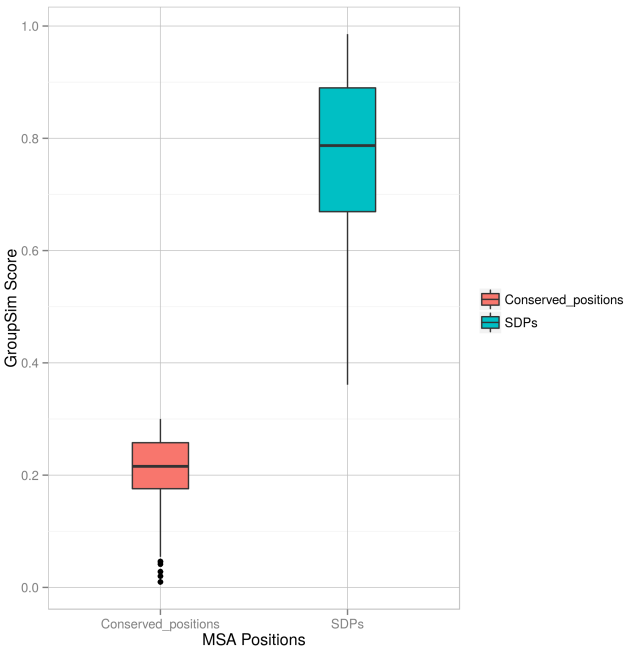

Supplementary Fig. 2: The range of GroupSim scores (G_s) for conserved positions and specificity-determining positions (SDPs) obtained for a benchmarking dataset by Chakraborty and Chakrabarti (2014).

3 SDP ratio (R_{sdp}) for inferring the Functional Coherence (FC) of a parent node MSA with two pre-defined child nodes.

To establish a suitable R_{sdp} ratio that ensures functional coherence for a pair of sequence clusters, we benchmarked ratios for a set of 30 superfamilies in order to distinguish between parent nodes having child nodes containing sequences that share the same EC and those containing different ECs. Optimal strategies for calculating the R_{sdp} ratio were determined depending on whether any of the child nodes had a low DOPS score or both the child nodes had high DOPS.

Supplementary Figure 3(a) shows that for parent nodes having any child node with low DOPS ($D_f = 0$), the $R_{sdp(lowDOPS)}$ ratio (Equation S1) tends to be negative for parent nodes sharing the same EC number and positive when the two groups have different EC numbers.

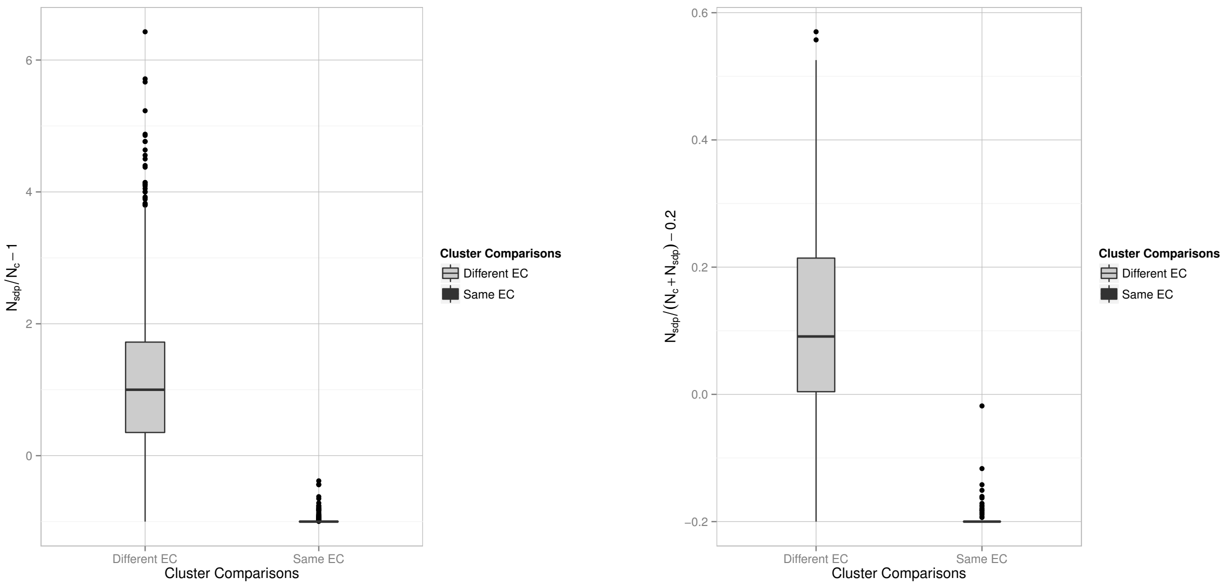

(a) $R_{sdp(lowDOPS)}$ (b) $R_{sdp(highDOPS)}$

Supplementary Fig. 3: R_{sdp} ratios used to distinguish between parent nodes containing two child nodes containing the same EC number and those containing different EC numbers when (a) one or both child nodes have low DOPS, and (b) both child nodes have high DOPS, for 200 functionally diverse CATH enzyme superfamilies which contains atleast two different EC4 numbers.

$$R_{sdp(lowDOPS)} = \frac{N_{sdp}}{N_c} - 1 \tag{S1}$$

Similarly, Supplementary Figure 3(b) shows that for parent clusters with both child nodes having high DOPS scores, the $R_{sdp(highDOPS)}$ ratio (Equation S2) also tends to be negative for parent nodes having child nodes sharing the same EC number in a MSA and positive when the two child nodes have different EC numbers.

$$R_{sdp(highDOPS)} = \frac{N_{sdp}}{N_c + N_{sdp}} - 0.2 \tag{S2}$$

Combining Equations S1 and S2, we get a generalized SDP Ratio (R_{sdp}):

$$R_{sdp} = D_f\,(\frac{N_{sdp}}{N_c + N_{sdp}} - 0.2) + (1 - D_f)(\frac{N_{sdp}}{N_c} - 1) \tag{S3}$$

where D_f is the DOPS factor of the MSA, N_{sdp} is the number of specificity-determining positions, N_c is the number of conserved positions in the MSA.

4 FunFHMMer algorithm workflow

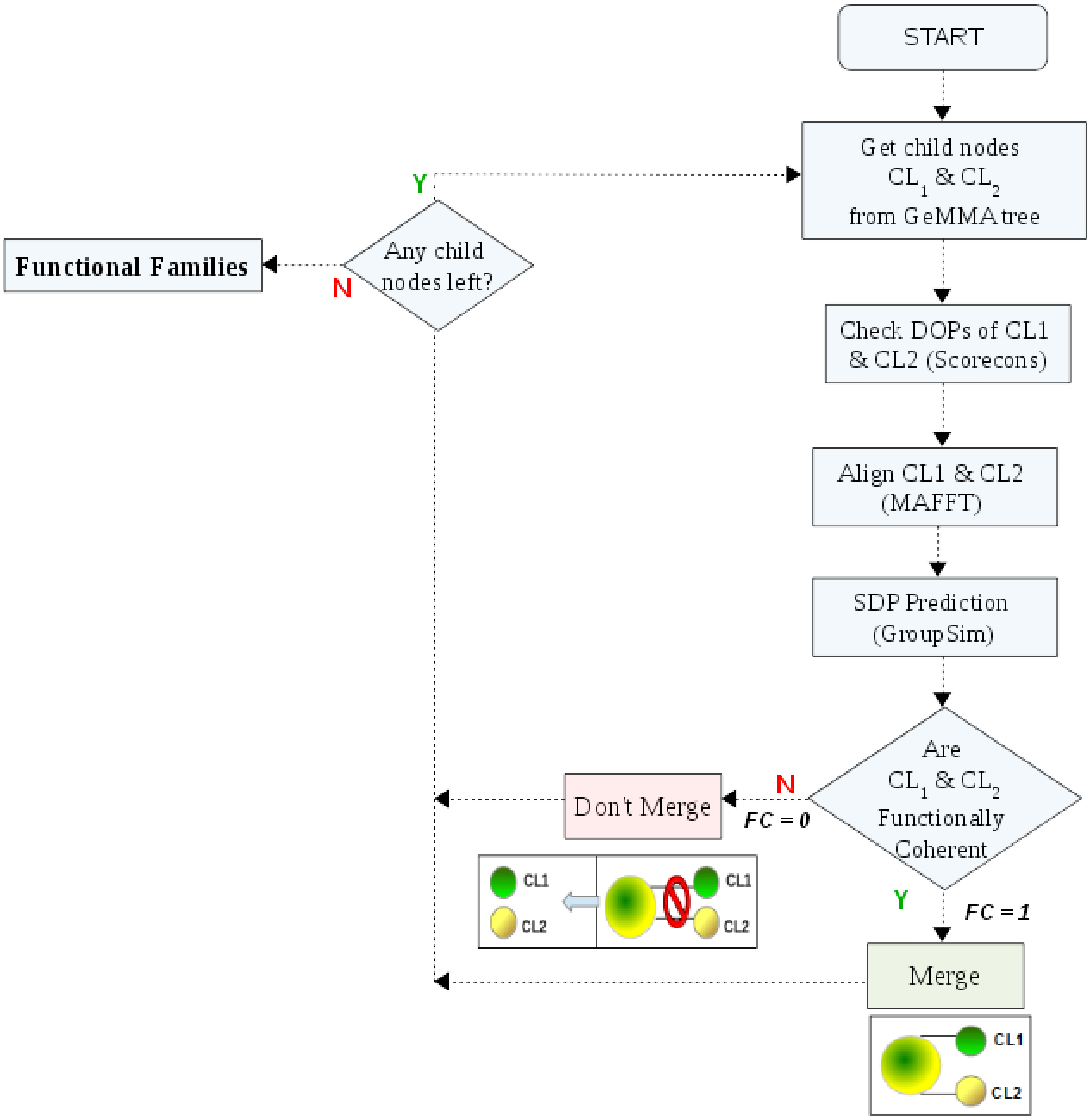

Supplementary Fig. 4: A flowchart outlining the FunFHMMer algorithm for assessing functional coherence of parent nodes in the hierarchical clustering tree.

5 UniProt Rollback Assessment

5.1 Depth of MFO annotations of the UniProt rollback assessment

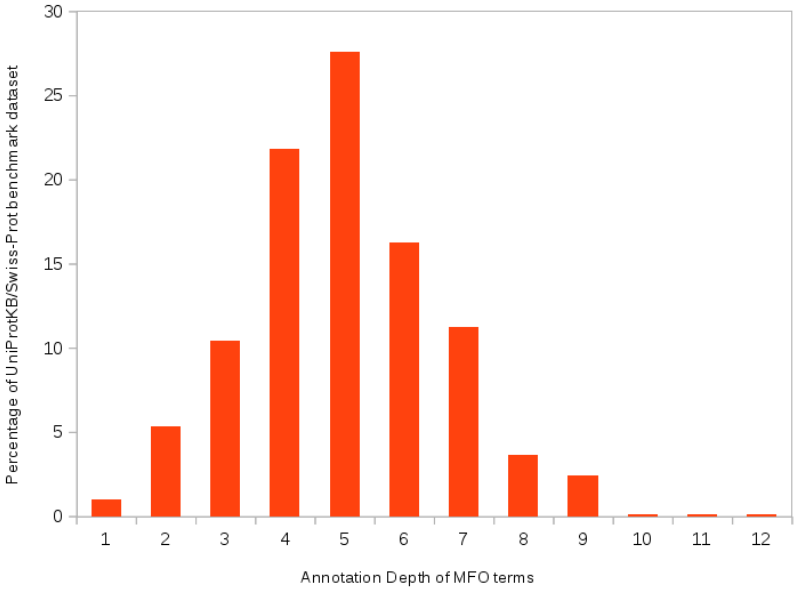

Supplementary Fig. 5: Distribution of depths of leaf term annotations of the UniProt rollback assessment proteins in Molecular Function Ontology (MFO).

5.2 Prediction of MFO annotations from Pfam/CDD family matches

5.2.1 Pfam family

The assessment proteins were scanned against the Pfam (version 27.0) (Finn *et al.*, 2014) family HMM models using HMMER3 (Eddy, 2010). The results were collapsed into a single set of Pfam domain architectures using DomainFinder3 (Yeats *et al.*, 2010) and regions on the proteins are assigned to a Pfam family if the E-value of the match to the HMM is significant (i.e. lower than the inclusion threshold of a Pfam family). The query sequences are assigned the high-quality MFO annotations (extracted from the UniProt-GOA annotation file dated May 28, 2013) of annotated sequences in the Pfam family, with a confidence score equal to the annotation frequency of the MFO term amongst all the annotated sequences of that family. This approach is similar to that used for assigning MFO terms and confidence scores to the CATH FunFam matches. The MFO annotations are then propagated up the MFO hierarchy or DAG and the final confidence scores associated with each MFO annotation after up-propagation.

5.2.2 CDD family

The assessment proteins were scanned against the CDD (version 3.10) (Marchler-Bauer *et al.*, 2014) family PSSM models using RPS-BLAST. The results were collapsed into a single set of CDD domain architectures using DomainFinder3 (Yeats *et al.*, 2010) and regions on the query proteins are assigned to a CDD family if the E-value of the match is significant (i.e. lower than the domain-specific score thresholds used by the NCBI CD-Search tool to determine whether hits to NCBI-curated domain models are specific or non-specific). The query sequences are assigned high-quality MFO annotations (extracted from the UniProt-GOA annotation file

dated May 28, 2013) of annotated sequences in the CDD family with a confidence score equal to the annotation frequency of the MFO term amongst all the annotated sequences of that family. This is similar to the approach used for Pfam family and CATH FunFam matches. The MFO annotations are then propagated up the MFO hierarchy or DAG and the final confidence scores associated with each MFO annotation after up-propagation (described below).

5.3 Evaluation metrics

As in CAFA (Radivojac *et al.*, 2013), for each target and some decision threshold $\tau \in [0,1]$, the GO terms in the Molecular Function Ontology (MFO) terms assigned to it with confidence scores greater than or equal to τ, were propagated up the MFO hierarchy or directed acyclic graph (DAG) to the root, yielding the set of predicted MFO terms for that target (predicted set). The true MFO terms were extracted from the November 28, 2013 UniProt-GOA file and were also up-propagated the MFO hierarchy for every target (true set). Any terms which overlap between the predicted and the true set were considered as correct at that decision threshold τ. As a result, the precision pr and recall rc for each target were computed as

$$pr_i(\tau) = \frac{\sum_f I(f \in P_i(\tau) \ \wedge \ f \in T_i)}{\sum_f I(f \in P_i(\tau))} \tag{S4}$$

$$rc_i(\tau) = \frac{\sum_f I(f \in P_i(\tau) \ \wedge \ f \in T_i)}{\sum_f I(f \in T_i)} \tag{S5}$$

where $I(f)$ is the standard indicator function, f is a MFO term, T_i is the set of true MFO terms (true set) for protein i and $P_i(\tau)$ is the set of predicted MFO terms for protein i with confidence score greater than or equal to τ. f ranges over the MFO hierarchy, excluding the root MFO term (GO:0003674). The precision-recall space was then generated by averaging precision and recall across all targets at a given threshold. The average precision and recall at a fixed threshold τ were calculated as

$$pr(\tau) = \frac{1}{m(\tau)} . \sum_{i=1}^{m(\tau)} pr_i(\tau) \tag{S6}$$

$$rc(\tau) = \frac{1}{n} . \sum_{i=1}^{n} rc_i(\tau) \tag{S7}$$

where n is the total number of targets, $m(\tau)$ is the number of targets $\leq n$, on which at least one prediction has been made above threshold τ.

Each prediction model was characterized by a precision-recall curve $(pr(\tau), rc(\tau))_\tau$. In order to use a single evaluation metric to compare the performance of different methods, the maximum F-measure (F_{max}, a harmonic mean between precision and recall, which gives equal emphasis to both) was used over all thresholds. It was calculated as,

$$F_{max} = max \ \tau \{ \frac{2.pr(\tau).rc(\tau)}{pr(\tau) + rc(\tau)} \} \tag{S8}$$

such that a perfect function prediction method would be characterized with F_{max} =1.

The coverage of predictions made by the methods were calculated by:

$$Coverage(C) = \frac{p}{N} * 100 \tag{S9}$$

where p is the number of sequences with at least 1 prediction by a method and N is the total number of sequences in the benchmark.

5.4 Comparison of performance in predicting function for hard targets

As many targets in the UniProt rollback assessment had very close homologues with functional annotations, which could be easily be recognised by the methods, we also checked the performance of the methods on a subset of the proteins in the dataset which are very hard, i.e. which do not have any functionally annotated relatives with sequence identity $>$ 50%. This dataset comprised of 553 proteins. Figure 6 shows the performance of FunFHMMer (F_{max} = 0.651, C = 62%), CDD (F_{max} = 0.575, C = 78%), Pfam (F_{max} = 0.555, C = 90%) and DFX (F_{max} = 0.553, C = 72%) on the hard targets of the Uniprot rollback assessment Dataset. It can be seen that similar to the relative performance of the methods is similar to the performance on the whole assessment set (see Figure 4).

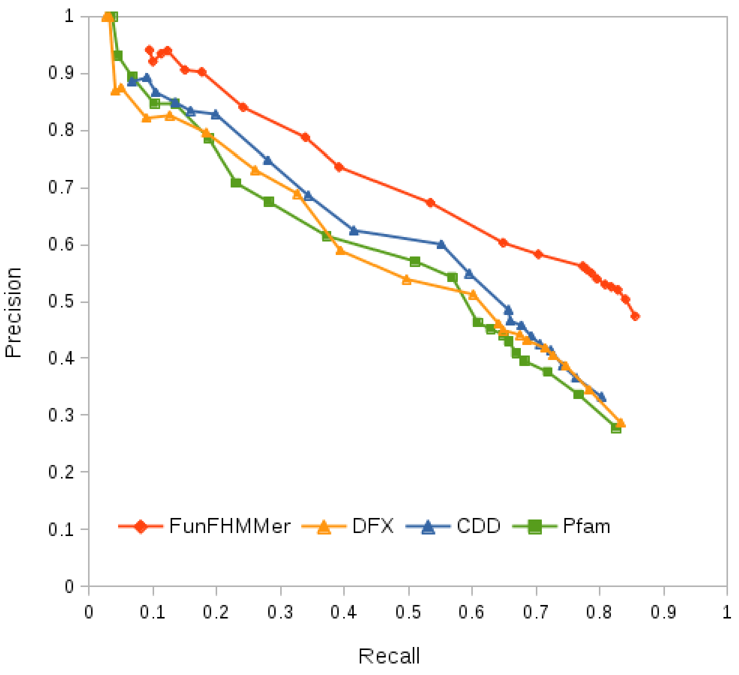

Supplementary Fig. 6: Performance of FunFHMMer protocol on hard targets in the UniProt rollback assessment dataset compared to functional annotations predicted by DFX protocol, Pfam family and CDD family assignments.

6 Residue Enrichment Analysis

A comparison of enrichment scores for a subset of FunFams, generated by FunFHMMer and DFX and sharing the same structural domains, showed that the conserved positions in FunFams generated by both are highly enriched in catalytic residues (p-value $< 4.31E{-}16$ for FunFHMMer and p-value $< 3.41E{-}15$ for DFX). However, a higher proportion of FunFHMMer FunFams were found to have all known catalytic residues conserved compared to DFX (Figure 7).

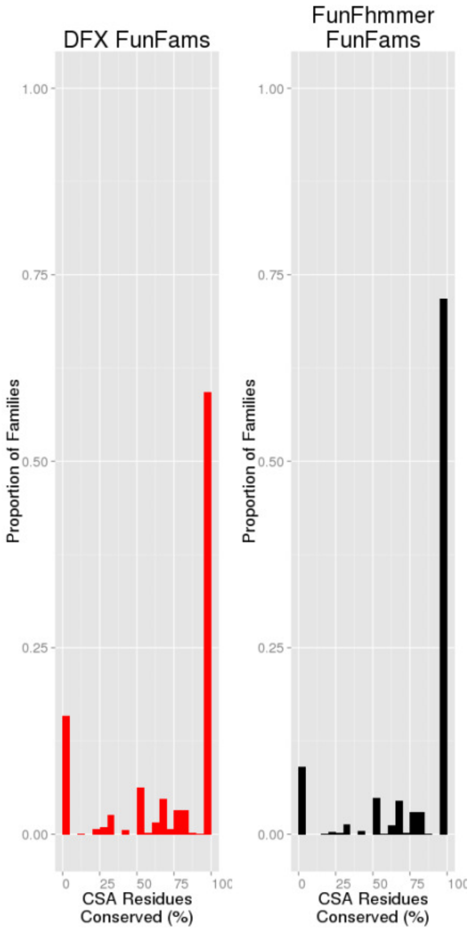

Supplementary Fig. 7: Comparison of the percentage of catalytic residues that are conserved in FunFams generated by DFX and FunFHMMer.

7 Structure-Function Linkage Database (SFLD) benchmark

The Structure-Function Linkage Database (SFLD) provides a manually-curated, "gold standard" set of mechanistically diverse enzyme superfamilies (Brown *et al.*, 2006) classified into families according to experimental data and serves as a challenging benchmark for validation of superfamily classification methods (Brown *et al.*, 2007; Lee *et al.*, 2010).

The benchmark sequences were taken from the SFLD on 24 February 2014. Although, the database currently contains 12 'gold-standard' core superfamilies, only 9 have been classified into families, namely Amidohydrolase, Crotonase, Enolase, Haloacid dehalogenase, Isoprenoid Synthase Type I, Isoprenoid Synthase Type II, Nucleophilic Attack 6-Bladed Beta-Propeller (N6P), Radical SAM and Rubisco. CATH-Gene3D domain superfamilies could be mapped onto these 9 SFLD superfamilies and a new dataset called the 'SFLD-Gene3D benchmark' dataset was created comprising all the CATH-Gene3D predicted sequences mapped to SFLD whole proteins. 7 of these SFLD superfamilies were single domain and were mapped to single CATH superfamilies. However, the Enolase and Rubisco superfamilies contained multi-domain proteins and were each mapped to two CATH superfamilies giving a total of 11 SFLD-Gene3D superfamilies which constituted the benchmarking dataset (see Supplementary Table 1). These superfamilies were then classified into FunFams by both DFX and FunFHMMer.

Supplementary Table 1: Composition of the SFLD and corresponding SFLD-Gene3D benchmark dataset

**SFLD superfamily**	**CATH-Gene3D superfamily**	**SFLD-Gene3D mapping (%)**
Amidohydrolase	3.20.20.140	99.7
Crotonase	3.90.226.10	100
Enolase	3.20.20.120	100
Enolase	3.30.390.10	99.6
Haloacid dehalogenase	3.40.50.1000	96.7
Isoprenoid Synthase Type I	1.10.600.10	99.6
Isoprenoid Synthase Type II	1.50.10.20	100
N6P	2.120.10.30	100
Radical SAM	3.20.20.70	67.7
Rubisco	3.20.20.110	100
Rubisco	3.30.70.150	100

Evaluation measures

The three classification evaluation measures used: purity, edit distance and VI distance were introduced by Brown *et al.* (2007) while the performance score was that used by Lee *et al.* (2010). The following equations and large parts of the explanatory text are thus directly taken from these publications:

Purity. Purity is defined as the percentage of families within which all annotated members are annotated with the same function. It is calculated as:

$$Purity(p) = \frac{Number\ of\ pure\ families}{Total\ number\ of\ families} \cdot 100 \qquad (S10)$$

Edit distance. Edit distance can be defined as the number of split or merge operations that are required to transform the predicted families into the families that correspond to the experimental functional annotations. The edit distance between the true partition (S) and the predicted partition (S') with families k and k' respectively, is calculated as:

$$Edit\ distance(e) \ = 2\{\sum_{k,k'} r_{k,k'}\} - K - K' \tag{S11}$$

where $r_{k,k'} \ = \ 1$ if families have sequences in common otherwise $r_{k,k'} \ = \ 0$ and K and K' are the total number of families in S and S'.

VI distance. VI distance can be defined as the amount of information not shared between the predicted families and the experimentally annotated families. The VI distance between S and S' is calculated as,

$$VI\ distance(v) = H(S) + H(S') - 2I(S, S') \tag{S12}$$

where H is the entropy of a partition and I is the mutual information between two partitions,

$$H(S) = \sum_{k=1}^{K} \frac{n_k}{N} log \frac{n_k}{N} \tag{S13}$$

$$I(S, S') = \sum_{k=1}^{K} \sum_{k'=1}^{K} \frac{n_{k,k'}}{N} log \frac{n_{k,k'}}{N} \tag{S14}$$

In Equations S13 and S14, n_k is the number of items in the family k of partition S, $n_{k,k'}$ is the number of overlapping items between the family k in partition S and the family k' in partition S' , and N is the total number of items in the set. Identical partitions will have both an edit and VI distance of zero.

Performance Score. The performance of the family-identification protocols on the SFLD superfamilies benchmark were compared using a performance score (Lee *et al.*, 2010) incorporating three distinct measures described above. The performance score (range 0-100) is then calculated as,

$$Performance\ score = \frac{2p \ + \ (100 - c_e.e) \ + \ (100 - c_v.v)}{4} \tag{S15}$$

where e_0 and v_0 are the initial values of edit and VI distance respectively and $c_e = \frac{100}{e_0}$, $c_v = \frac{100}{v_0}$.

Performance of FunFHMMer and DFX

The performance of FunFHMMer and DFX protocol on the SFLD-Gene3D benchmark dataset can be seen in Figure 8. FunFHMMer outperforms DFX on the SFLD-Gene3D benchmark set on average except in the Isoprenoid Synthase Type I superfamily (CATH 1.10.600.10) where both show poor performance. The Isoprenoid Synthase Type I superfamily have a large number of sequences that have no functional annotations which makes any study relating to function in this particular superfamily very challenging (Brown and Babbitt, 2014).

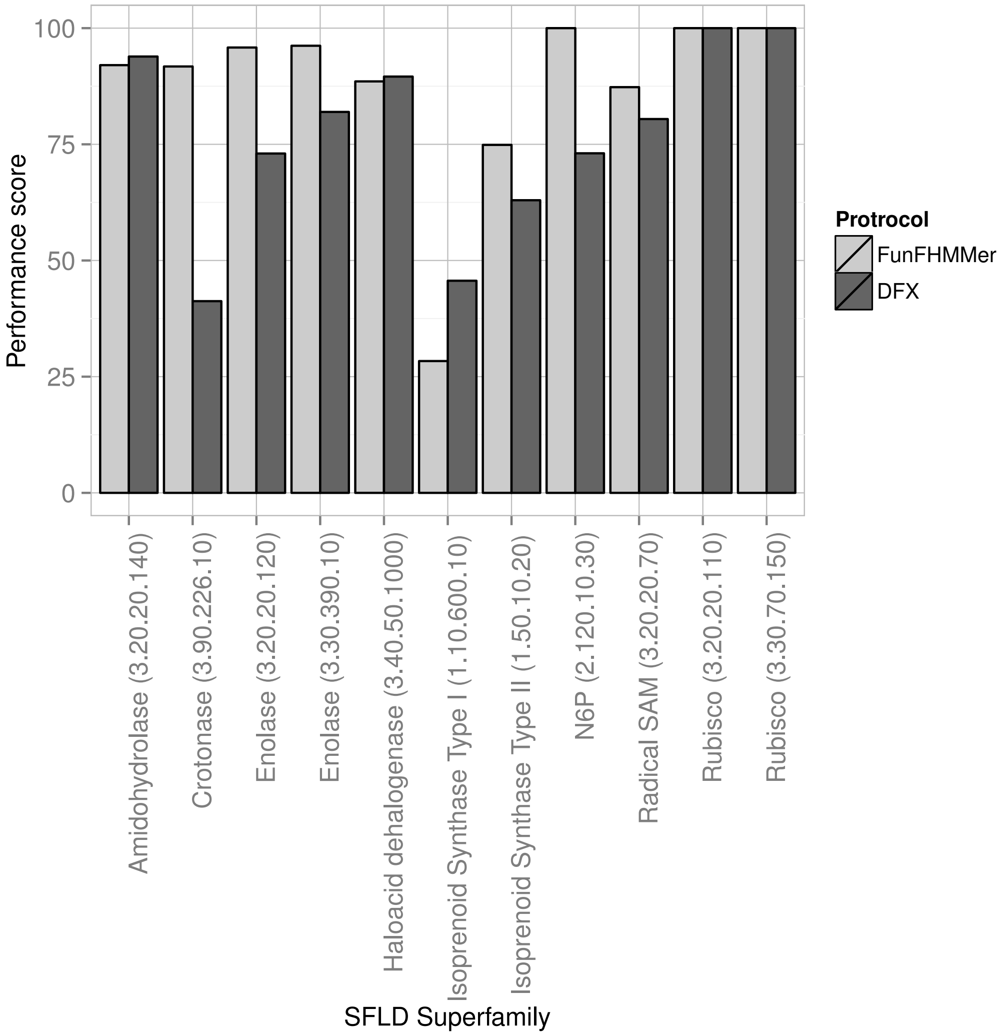

Supplementary Fig. 8: Performance of FunFHMMer and DFX protocol on the SFLD-Gene3D benchmark dataset.

References

Brown, D. P., Krishnamurthy, N., and Sjölander, K. (2007). Automated protein subfamily identification and classification. *PLoS computational biology*, **3**(8), e160.

Brown, S. D. and Babbitt, P. C. (2014). New insights about enzyme evolution from large scale studies of sequence and structure relationships. *Journal of Biological Chemistry*, **289**(44), 30221–30228.

Brown, S. D., Gerlt, J. A., Seffernick, J. L., and Babbitt, P. C. (2006). A gold standard set of mechanistically diverse enzyme superfamilies. *Genome biology*, **7**(1), R8.

Chakrabarti, S., Bryant, S. H., and Panchenko, A. R. (2007). Functional specificity lies within the properties and evolutionary changes of amino acids. *Journal of molecular biology*, **373**(3), 801–810.

Chakraborty, A. and Chakrabarti, S. (2014). A survey on prediction of specificity-determining sites in proteins. *Briefings in Bioinformatics*.

Eddy, S. (2010). Hmmer3: a new generation of sequence homology search software. url: http://hmmer. janelia. org.

Finn, R. D., Bateman, A., Clements, J., Coggill, P., Eberhardt, R. Y., Eddy, S. R., Heger, A., Hetherington, K., Holm, L., Mistry, J., *et al.* (2014). Pfam: the protein families database. *Nucleic acids research*, **42**(D1), D222–D230.

Lee, D. A., Rentzsch, R., and Orengo, C. (2010). Gemma: functional subfamily classification within superfamilies of predicted protein structural domains. *Nucleic acids research*, **38**(3), 720–737.

Marchler-Bauer, A., Derbyshire, M. K., Gonzales, N. R., Lu, S., Chitsaz, F., Geer, L. Y., Geer, R. C., He, J., Gwadz, M., Hurwitz, D. I., *et al.* (2014). Cdd: Ncbi's conserved domain database. *Nucleic acids research*, page gku1221.

Radivojac, P., Clark, W. T., Oron, T. R., Schnoes, A. M., Wittkop, T., Sokolov, A., Graim, K., Funk, C., Verspoor, K., Ben-Hur, A., *et al.* (2013). A large-scale evaluation of computational protein function prediction. *Nature methods*, **10**(3), 221–227.

Yeats, C., Redfern, O. C., and Orengo, C. (2010). A fast and automated solution for accurately resolving protein domain architectures. *Bioinformatics*, **26**(6), 745–751.
